# Non-toxic engineered carbon nanodiamond concentrations induce oxidative/nitrosative stress, imbalance of energy metabolism, and mitochondrial dysfunction in microglial and alveolar basal epithelial cells

**DOI:** 10.1038/s41419-018-0280-z

**Published:** 2018-02-14

**Authors:** Claudia G. Fresta, Aishik Chakraborty, Manjula B. Wijesinghe, Angela M. Amorini, Giacomo Lazzarino, Giuseppe Lazzarino, Barbara Tavazzi, Susan M. Lunte, Filippo Caraci, Prajnaparamita Dhar, Giuseppe Caruso

**Affiliations:** 1 Ralph N. Adams Institute for Bioanalytical Chemistry, University of Kansas, 66045 Lawrence, KS USA; 2 Department of Pharmaceutical Chemistry, University of Kansas, 66045 Lawrence, KS USA; 3 Department of Chemical and Petroleum Engineering, University of Kansas, 66045 Lawrence, KS USA; 4 Institute of Biochemistry and Clinical Biochemistry, Catholic University of the Sacred Heart, 00168 Rome, Italy; 5 Department of Biomedical and Biotechnological Sciences, Division of Medical Biochemistry, University of Catania, 94018 Catania, Italy; 6 Department of Chemistry, University of Kansas, 66045 Lawrence, KS USA; 7 Oasi Research Institute - IRCCS, 94018 Troina, Italy; 8 Department of Drug Sciences, University of Catania, 95125 Catania, Italy

## Abstract

Engineered nanoparticles are finding a wide spectrum of biomedical applications, including drug delivery and capacity to trigger cytotoxic phenomena, potentially useful against tumor cells. The full understanding of their biosafety and interactions with cell processes is mandatory. Using microglial (BV-2) and alveolar basal epithelial (A549) cells, in this study we determined the effects of engineered carbon nanodiamonds (ECNs) on cell viability, nitric oxide (NO) and reactive oxygen species (ROS) production, as well as on energy metabolism. Particularly, we initially measured decrease in cell viability as a function of increasing ECNs doses, finding similar cytotoxic ECN effects in the two cell lines. Subsequently, using apparently non-cytotoxic ECN concentrations (2 µg/mL causing decrease in cell number < 5%) we determined NO and ROS production, and measured the concentrations of compounds related to energy metabolism, mitochondrial functions, oxido-reductive reactions, and antioxidant defences. We found that in both cell lines non-cytotoxic ECN concentrations increased NO and ROS production with sustained oxidative/nitrosative stress, and caused energy metabolism imbalance (decrease in high energy phosphates and nicotinic coenzymes) and mitochondrial malfunctioning (decrease in ATP/ADP ratio).

These results underline the importance to deeply investigate the molecular and biochemical changes occurring upon the interaction of ECNs (and nanoparticles in general) with living cells, even at apparently non-toxic concentration. Since the use of ECNs in biomedical field is attracting increasing attention the complete evaluation of their biosafety, toxicity and/or possible side effects both in vitro and in vivo is mandatory before these highly promising tools might find the correct application.

## Introduction

Nanotechnology is considered one of the most promising field of applied research which is worldwide receiving considerable attention even from the media. Nanotechnology is producing impressive improvements in different disciplines on a large scale, such as physics and engineering. Even in medicine, nanotechnology offers great promises for new strategies of delivering that involve the use of nano-sized particles (nanoparticles)^1^.

In the last decade, engineered nanoparticles have found a wide spectrum of applications that range from energy production^2^ to industrial production processes^3^ to biomedical applications^4, 5^. The latter includes drugs delivery to tumors^6–8^, break up clusters of bacteria enhancing bacterial killing^9^, stimulation of immune responses^10, 11^, improvement of non-invasive imaging methods^12^, and scavenging of reactive oxygen species (ROS)^13^. Even though engineered nanoparticles use is becoming indispensable in many areas of human activity the debate regarding their toxicity and other side effects is still open^14, 15^.

Among the various types of engineered nanoparticles currently under investigation, we focused our attention on the effect of carbon nanoparticles, specifically engineered carbon nanodiamonds (ECNs), on brain and lung cells. It has already been shown that ECNs are able to induce alterations in lipid mixture mimicking the cell plasma membranes as a function of phospholipid headgroup charge and alkyl chain saturation in vitro^16^. Many factors, including size and shape, can influence the activity and toxicity of carbon nanoparticles^17^. Since they are frequently employed in a broad array of industrial and scientific commercial products and might become more easily inhalable at different stages of their life cycle^18^, the probability for human being to get in close contact with them is considerably increasing^19, 20^.

It has been widely shown that nanoparticles and their agglomerates in the range size of 10−200 nm, after inspiration, are significantly accumulated in the alveolar regions of the lungs, interacting with a complex mixture of essential molecules, such as lipids, proteins, and carbohydrates, forming the so called lung surfactants (LS)^21^. Two of the most important functions of LS are to form the first line of defence against any foreign particles^22^ and to maintain a low surface tension in the lung thus preventing their collapse^23, 24^. However, since it is highly probable that inhaled ECNs are not confined in the respiratory tract, it is important to investigate the effect and toxicity of ECNs on cell systems representative of additional relevant human tissues other than lungs. To this purpose, it is also worth recalling that ECNs have also recently been studied as a novel potential drug delivery system for treatment of malignant brain gliomas^25^, as well as in neurodegenerative disorders such as Alzheimer's disease^26^.

In the present study, the influence of different concentrations of ECNs in absence or presence of LS (DPPC:POPG(7:3)) on A549 and BV-2 cell toxicity was firstly investigated. Human alveolar basal epithelial cells A549 were selected not only because the lung is a primary site of nanoparticles retention after inspiration^21^, but also because A549 cells represent a preference model to study toxicity mediated by ROS generation^27–29^. The brain microglial cells BV-2 were chosen since they represent a valid model system alternative to primary microglia cultures^30^, with which they share as a common feature the responses to inflammatory stimuli and trophic factors^31^.

Additionally, in order to understand the impact of ECNs on different cellular biochemical functions, we used selected non-cytotoxic ECNs concentration to determine its influence on nitric oxide (NO) and total ROS production, and of metabolites related to energy metabolism, mitochondrial functions, oxido-reductive reactions, and antioxidant defences.

## Results

### Effect of ECNs and DPPC:POPG(7:3)/ECNs on A549 and BV-2 cells toxicity

**Fig. 1:**
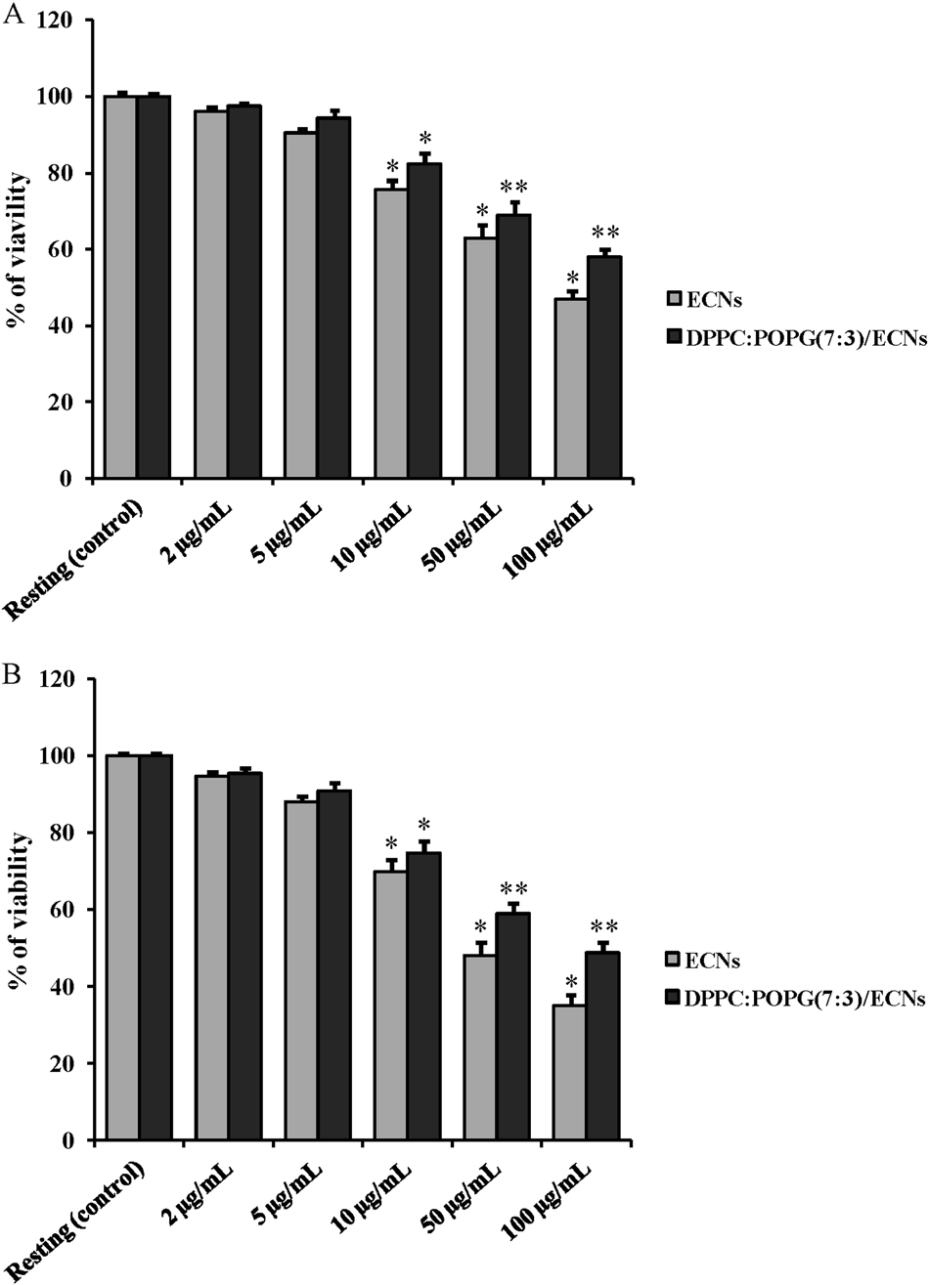
Change in the cell viability caused by challenging for 24 h A549 (Panel A) and BV-2 (Panel **B**) cells with increasing concentration (2, 5, 10, 50, or 100 µg/mL) of ECNs or DPPC:POPG(7:3)/ECNs. Data are the mean of four independent experiments and are expressed as the percent variation with respect to the viability recorded in control cultures. Standard deviations are represented by vertical bars. *Significantly different from resting (control) cells, *p* < 0.01; **Significantly different from corresponding concentration of ECNs in absence of DPPC:POPG(7:3), *p* < 0.01

The first aim of the present study was to investigate the toxicity of different concentrations of ECNs on A549 and BV-2 cells. This was done both in the absence and presence of lipids (DPPC:POPG(7:3)) that were used as model LS (biological) systems that are expected to coat ECNs that enter the body through the respiratory route. A representative picture at low (2 h after seeding) and high density for both A549 and BV-2 cells employed in our study is shown in Supplementary Figure 1. Incubation of A549 or BV-2 cells for 24 h with increasing concentration (2, 5, 10, 50, or 100 µg/mL) of ECNs provoked a dose-dependent increase in cell toxicity (Fig. 1A, B). Maximal decrease in cell viability was obtained when using 100 µg/mL ECNs (−53 and −65% in A549 or BV-2 cells, respectively, *p* < 0.01 compared to resting (untreated) cells). The presence of DPPC:POPG(7:3) in combination with ECNs produced significant protection only when both cell types were treated with high doses (50 and 100 µg/mL) of ECNs.

From this set of data, we were able to select the nanoparticle concentration (2 µg/mL) that produced a decrease in cell viability, in both A549 or BV-2 cells, lower than 5%. Additionally, we also excluded from further experiments the use of artificial LS since, at this selected ECNs concentration, no differences were recorded in cell viability in presence or absence of DPPC:POPG(7:3).

To further confirm the results on cell viability obtained with the MTT assay, we performed additional experiments measuring release of lactate dehydrogenase in the culture media, as an index of cell necrosis. Moreover, we also evaluated cell proliferation and survival using the XTT assay and the trypan blue test, respectively. Compared to resting cells, the treatment with 2 µg/mL ECNs for 24 h provoked an increase in LDH release in A549 (+4%) and BV-2 (+5%) cells (Supplementary Figure 2) that was similar in magnitude to the results of the MTT test (see Fig. 1). Additionally, values of cell proliferation and survival were very similar between resting and ECNs-treated cells (Supplementary Tables 1 and 2).

Supplementary Figure 3 shows the effect of longer times of cell exposition (48 and 72 h) to 2 µg/mL ECNs. As in the case of 24 h incubation, the decrease in viability observed in A549 and BV-2 cells was quite low for both incubation times compared to resting cells, with a resting/ECNs-treated cells ratio equal to 1.13 and 1.16 for A549 and BV-2 cells, respectively, after 72 h.

### Effect of ECNs on NO production of cultured lung and microglial cells

**Fig. 2:**
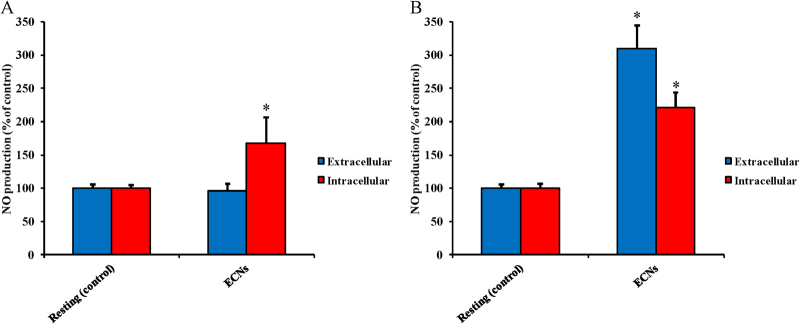
Intracellular and extracellular concentrations of NO (as determined by Griess assay) in resting and ECNs-stimulated (24 h) A549 (Panel A) and BV-2 (Panel **B**) cells. Data are the mean of four independent experiments. Standard deviations are represented by vertical bars. *Significantly different from resting (control) cells, *p* < 0.001

Figure 2 shows the effect of apparently non-cytotoxic ECN concentrations on the intracellular and extracellular NO production (as determined by Griess assay) in cultured A549 (Panel A) and BV-2 (Panel B) cells. The addition of ECNs to the culture medium of A549 for 24 h did not significantly affect the extracellular NO concentration but provoked a 68% increase in the intracellular NO concentration (*p* < 0.001 compared to control cells). Differently, microglial BV-2 cells underwent dramatic increase in both extracellular and intracellular NO generation. Particularly, 210 and 121% increase, respectively, in extracellular and intracellular NO production (*p* < 0.001 respect to control resting cells) was measured after 24 h incubation with a dosage of ECNs (2 μg/mL) that did not cause significant decrease in cell viability. The 1 h pre-treatment of A549 or BV-2 cells with L-NAME (500 µM) or L-MMA (1 mM), two well-known iNOS inhibitors, almost completely abolished the difference in NO production between resting and ECNs-treated cells (Supplementary Figure 4).

### ROS production induced by non-toxic ECNs concentration in cultured lung and microglial cells

**Fig. 3:**
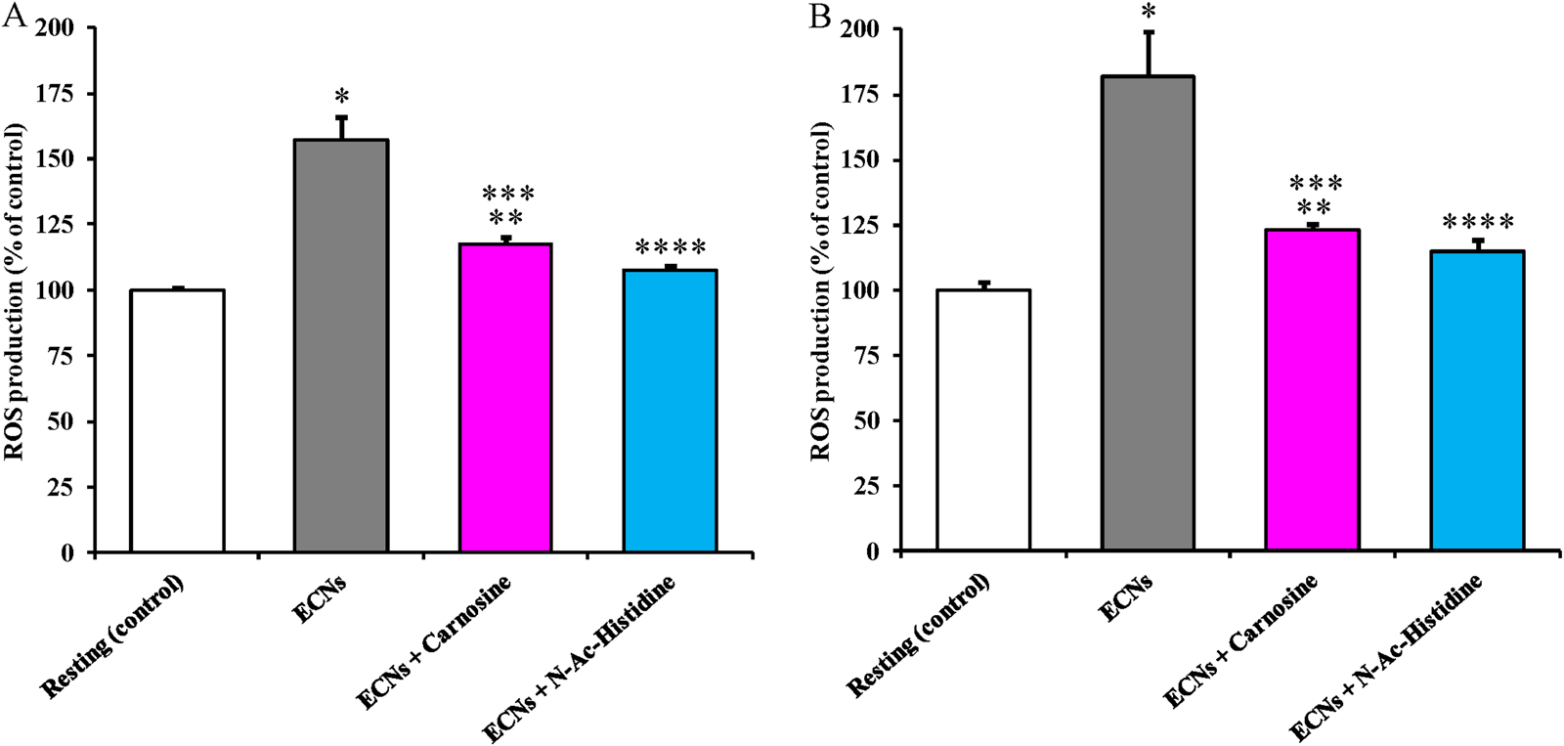
Total ROS production (as detected by microchip electrophoresis with laser-induced fluorescence) in resting and ECNs-stimulated (24 h) A549 **(**Panel **A**) and BV-2 (Panel **B)** cells. Carnosine and *N*-acetyl-L-histidine (N-Ac-Histidine) (10 mM) are pre-treatments (1 h). Data are the mean of 4 independent experiments and are expressed as the percent variation with respect to the total ROS production recorded in control cultures. Standard deviations are represented by vertical bars. *Significantly different from resting (control) cells, *p* < 0.001; **Significantly different from resting (control) cells, *p* < 0.01; ***Significantly different from ECNs-treated cells, *p* < 0.01; ****Significantly different from ECNs-treated cells, *p* < 0.001

Results illustrated in Fig. 3 demonstrate that a non-toxic ECNs concentration is capable to cause a 57 and 83% increase in total ROS production (as determined by ME-LIF) in cultured A549 (Panel A) and BV-2 (Panel B) cells, respectively (*p* < 0.001 compared to resting cells). To counteract ECNs-mediated ROS overproduction, we tested the effect of 10 mM *N*-acetyl-L-histidine or carnosine by pre-treating both cell lines for 1 h before their challenge with ECNs at 2 μg/mL. Either in lung or microglial cells, *N*-acetyl-L-histidine was more effective than carnosine in decreasing ECNs-induced ROS generation. In fact, respect to control resting cells, a still 20% higher ROS formation was measured in ECNs-carnosine treated cells (*p* < 0.01 respect to both resting and ECNs-treated cells), whilst ECNs-*N*-acetyl-L-histidine treated cells had levels of ROS not significantly different from those measured in resting cells (*p* < 0.001 compared to ECNs-treated cells).

To investigate the observed effect on ROS production by cultured A549 and BV-2 cells in the presence of *N*-acetyl-L-histidine or carnosine, we performed additional experiments in which ROS production was measured after incubating the cells for 24 h with ECNs following a 1 h pre-incubation with β-alanine, L-histidine, or β-alanine + L-histidine (all at 10 mM). In stimulated A549 and BV-2 cells, neither β-alanine nor L-histidine nor their equimolar combination induced a significant decrease in ROS production if compared to the treatment with carnosine or *N*-acetyl-L-histidine (data not shown).

### Influence of non-toxic ECNs concentration on energy metabolism, mitochondrial functions, nicotinic coenzymes, and oxidant/antioxidant balance of cultured lung and microglial cells

Table 1 summarizes results of the effect of the addition to the culture medium for 24 h of non-toxic ECNs amount, on parameters related to energy metabolism and mitochondrial functions of cultured A549 and BV-2 cells. In both cell lines, the energy state, evaluated in terms of concentrations of ATP, ADP, AMP, and energy charge potential (ECP = ATP + 1/2 ADP/ATP + ADP + AMP) was negatively affected by 2 μg/mL ECNs. Significant −23 and −29% decrease of ATP in A549 and BV-2 cells, respectively, (*p* < 0.001 compared to resting cells) was accompanied by modest increase in ADP and an impressive increase in AMP (+338 and +418%, respectively, in A549 and BV-2 cells; *p* < 0.001 compared to resting cells). The imbalance in adenine nucleotide homeostasis in both cell lines, consequently caused significant decrease in ECP (*p* < 0.001 compared to resting cells), which is a good measure of the cell energy state. Using the values of the concentrations of ATP and ADP (expressed as nmol/mg protein), it was possible to calculate the ATP/ADP ratio, which is considered as a reliable index of the mitochondrial phosphorylating capacity^32^. A549 and BV-2 cells showed decrease in the ATP/ADP ratio by −32 and −58%, respectively, (*p* < 0.001 compared to resting cells) indicating either that non-toxic levels of ECNs deeply alter the main mitochondrial function, i.e., to ensure adequate energy supply to the cell, or that microglial cells are more sensitive to the negative effects induced on cell metabolism by ECNs.

**Table 1:** Effect of 24 h of incubation of alveolar basal epithelial A549 and microglial BV-2 cells with engineered carbon nanodiamonds (ECNs) on adenine nucleotides (mono, di, and triphosphorylated), cell energy state (ECP) and mitochondrial phosphorylating capacity (ATP/ADP ratio)

	ATP (nmol/mg protein)	ADP (nmol/mg protein)	AMP (nmol/mg protein)	ECP	ATP/ADP
Resting A549	95.07 (19.02)	8.25 (2.03)	1.24 (0.34)	0.96 (0.11)	11.52 (1.12)
A549 + ECNs	72.73* (14.58)	9.34** (1.18)	4.19* (0.72)	0.89* (0.12)	7.79* (0.65)
Resting BV-2	123.90 (29.68)	15.93 (2.35)	2.07 (0.62)	0.93 (0.07)	7.78 (1.12)
BV-2 + ECNs	87.87* (17.42)	26.83* (5.65)	8.66* (1.51)	0.82* (0.11)	3.28* (0.94)

Values are the mean of four different experiments. Standard deviations are in parenthesis. Incubation conditions and HPLC method to separate simultaneously the indicated compounds are given in Materials and methods

*ECNs* engineered carbon nanodiamonds, *ATP*, *ADP*, *AMP* adenosine mono, di, triphosphate, respectively; *ECP* energy charge potential (ATP + 1/2 ADP/ATP + ADP + AMP)

*Significantly different from resting, *p* < 0.001; **Significantly different from resting, *p* < 0.05

Results reported in Table 2 strongly demonstrate that the ECNs concentration non-toxic for cell viability (2 μg/mL) was however capable to deeply affect concentrations of purine (GTP, GDP, GMP, IMP) and pyrimidine (UTP, UDP, UMP, CTP, CDP, CMP) nucleotides, causing an overall dramatic depletion of the cellular stores of high energy phosphates (each of these compounds was significantly different from resting cells in both A549 and BV-2, *p* < 0.01).

**Table 2:** Effect of 24 h of incubation of alveolar basal epithelial A549 and microglial BV-2 cells with engineered carbon nanodiamonds (ECNs) on purine and pyrimidine nucleotides (mono, di, and triphosphorylated)

	GTP (nmol/mg protein)	GDP (nmol/mg protein)	GMP (nmol/mg protein)	IMP (nmol/mg protein)	UTP (nmol/mg protein)	UDP (nmol/mg protein)	UMP (nmol/mg protein)	CTP (nmol/mg protein)	CDP (nmol/mg protein)	CMP (nmol/mg protein)
Resting A549	25.71 (8.44)	4.51 (1.13)	0.64 (0.10)	0.40 (0.05)	39.56 (4.05)	0.58 (0.09)	0.18 (0.04)	26.32 (3.47)	0.52 (0.12)	0.047 (0.018)
A549 + ECNs	16.14* (2.07)	5.69* (1.38)	0.96* (0.12)	0.49* (0.07)	32.73* (3.71)	1.20* (0.38)	0.16* (0.03)	20.10* (2.21)	0.76* (0.17)	0.043* (0.011)
Resting BV-2	21.79 (5.13)	6.73 (2.02)	2.05 (0.79)	2.56 (0.31)	45.83 (6.25)	3.09 (0.89)	0.77 (0.26)	16.34 (3.32)	2.45 (0.68)	1.37 (0.45)
BV-2 + ECNs	17.32* (3.37)	6.01* (1.51)	4.85* (0.74)	2.28* (0.56)	33.36* (8.13)	4.58* (1.64)	0.59* (0.28)	10.92* (2.45)	1.95* (0.72)	1.32* (0.19)

Values are the mean of four different experiments. Standard deviations are in parenthesis. Incubation conditions and HPLC method to separate simultaneously the indicated compounds are given in Materials and methods

*ECNs* engineered carbon nanodiamonds, *GTP*, *GDP*, *GMP* guanosine mono, di, triphosphate, respectively; *IMP* inosine monophosphate, *UTP*, *UDP*, *UMP* uridine mono, di, triphosphate, respectively; *CTP*, *CDP*, *CMP* cytidine mono, di, triphosphate, respectively

*Significantly different from resting, *p* < 0.01

Data referring to oxidized (NAD^+^ and NADP^+^) and reduced (NADH and NADPH) nicotinic coenzymes in resting A549 and BV-2, and in cells challenged with a non-toxic ECNs concentration are summarized in Table 3. ECNs at 2 μg/mL produced a significant decrease in NAD^+^ and NADP^+^ concentrations in both cell lines (*p* < 0.01 compared to resting cells), with concomitant increase in NADH and decrease in NADPH. In consequence of these changes, either the NAD^+^/NADH or the NADP^+^/NADPH ratio were modified by ECNs. A −44 and −30% decrease of NAD^+^/NADH and NADP^+^/NADPH ratio, respectively, was observed in A549 treated cells (*p* < 0.001 in comparison with resting cells), while −35 and −13% of these values were found in BV-2 treated cells (*p* < 0.001 compared to resting cells). It should also be considered that the total pool of nicotinic coenzymes (NAD^+^ + NADH + NADP^+^ + NADPH) was 22.56 nmol/mg protein in resting A549 and 39.07 nmol/mg protein in resting BV-2. In ECNs-stimulated A549 and BV-2 cells, these values were 17.61 (−22%, *p* < 0.001 compared to resting cells) and 31.09 nmol/mg protein (−20%, *p* < 0.001 compared to resting cells), respectively, indicating an equal depletion of the total nicotinic coenzyme pool induced by the non-cytotoxic ECNs treatment.

**Table 3:** Effect of 24 h of incubation of alveolar basal epithelial A549 and microglial BV-2 cells with engineered carbon nanodiamonds (ECNs) on oxidized and reduced nicotinic coenzymes

	NAD^+^ (nmol/mg protein)	NADH (nmol/mg protein)	NADP^+^ (nmol/mg protein)	NADPH (nmol/mg protein)	NAD^+^/NADH	NAPD^+^/NAPDH
Resting A549	16.92 (4.28)	1.25 (0.48)	3.96 (0.94)	0.43 (0.05)	13.54 (1.87)	9.21 (1.29)
A549 + ECNs	13.77* (2.59)	1.83* (0.56)	1.74* (0.62)	0.27* (0.07)	7.52* (1.05)	6.44* (0.65)
Resting BV-2	29.88 (2.32)	2.95 (0.30)	5.53 (0.82)	0.71 (0.06)	10.13 (1.55)	7.79 (0.84)
BV-2 + ECNs	23.04* (5.24)	3.52* (0.61)	3.95* (0.91)	0.58* (0.04)	6.54* (0.94)	6.81* (0.72)

Values are the mean of four different experiments. Standard deviations are in parenthesis. Incubation conditions and HPLC method to separate simultaneously the indicated compounds are given in Materials and methods

*ECNs* engineered carbon nanodiamonds, *NAD*^*+*^ oxidized nicotinamide adenindinucleotide, *NADH* reduced nicotinamideadenindinucleotide, *NADP*^*+*^ oxidized nicotinamideadenindinucleotide phosphate, *NADH* reduced nicotinamideadenindinucleotide phosphate

*Significantly different from resting, *p *< 0.001

In Table 4, the values of reduced glutathione (GSH), the water-soluble low-molecular weight antioxidant protecting free-SH groups of proteins, of malondialdehyde (MDA), an end-product of ROS mediated peroxidation of fatty acids of membrane phospholipids, and of nitrite and nitrate, the end-products of NO metabolism, are summarized. Significant decrease by 59 and 61% respect to resting cells (*p* < 0.001) of GSH was recorded in ECNs-treated A549 and BV-2, respectively. Concomitantly, three times higher MDA values were measured in both stimulated cell lines (*p* < 0.001 compared to resting cells) being suggestive of significant ROS mediated damages to biological membranes. Intracellular nitrite and nitrate (measured by HPLC using a method for their direct detection, with no derivatization) were statistically higher in ECNs treated cells (*p* < 0.001 compared to resting cells). The nitrite + nitrate sum was 6.61 and 12.63 nmol/mg protein in resting A549 and BV-2 cells, respectively. Values of this sum in cells treated with non-toxic ECNs were 10.83 (+64%) and 23.22 (+84%) nmol/mg protein (*p* < 0.001 compared to resting cells), indirectly indicating a remarkable increase in NO production during the 24 h challenge with ECNs.

**Table 4:** Effect of 24 h of incubation of alveolar basal epithelial A549 and microglial BV-2 cells with engineered carbon nanodiamonds (ECNs) on GSH and parameters related to oxidative (MDA) and nitrosative stress (nitrite, nitrate)

	GSH (nmol/mg protein)	MDA (nmol/mg protein)	Nitrite (nmol/mg protein)	Nitrate (nmol/mg protein)
Resting A549	0.49 (0.09)	0.008 (0.002)	0.04 (0.01)	6.57 (0.85)
A549 + ECNs	0.20* (0.04)	0.025* (0.008)	0.17* (0.05)	10.66* (1.89)
Resting BV-2	0.38 (0.06)	0.010 (0.003)	0.11 (0.03)	12.52 (5.77)
BV-2 + ECNs	0.15* (0.05)	0.031* (0.007)	0.31* (0.07)	22.91* (7.06)

Values are the mean of four different experiments. Standard deviations are in parenthesis. Incubation conditions and HPLC method to separate simultaneously the indicated compounds are given in Materials and methods

* ECNs* engineered carbon nanodiamonds, *GSH* reduced glutathione, *MDA* malondialdehyde

*Significantly different from resting, *p* < 0.001.

## Discussion

The use of nanoparticles in industry, biology, and medicine is attracting increasing attention. In particular carbon nanoparticles have been recently considered as new emerging tools for delivery systems for cancer therapy and have contributed to therapeutic strategies against different neurological disorders^33^ including Alzheimer's disease^34, 35^. Therefore, several studies have been aimed in evaluating their biosafety, toxicity, and/or possible side effects both in vitro and in vivo^36^. These information are of paramount importance to finalize the use of nanoparticles for therapeutic applications (e.g., drug delivery). Notwithstanding, their effects on cell metabolism have not yet been fairly described. Particularly, no results have been to date produced on the influence of non-cytotoxic concentrations of ECNs on cell metabolism and mitochondrial functions.

The experiments described in the present study were purposely designed in order to determine, in two different alveolar basal epithelial A549 and brain microglial BV-2 cell lines, whether non-cytotoxic amount of ECN was capable to cause increase in NO and ROS production and to induce significant modifications in mitochondrial functions and energy metabolism. Results indicate that: (i) ECNs caused a dose-dependent decrease in cell viability of A549 or BV-2 cells, with maximal decrease obtained when using 100 µg/mL ECNs; (ii) the presence of lipid mixtures (DPPC:POPG(7:3)) mimicking the composition of LS provided additional protections against ECNs toxicity only at very high ECN concentrations (50 and 100 µg/mL); (iii) non-cytotoxic ECN concentrations were able to increase both total NO (intracellular + extracellular) and ROS production in both cell lines; (iv) nanoparticle treatment of A549 and BV-2 cells negatively affected concentrations of high-energy phosphates, nicotinic coenzymes, and GSH, indicating imbalance of energy metabolism and mitochondrial functions and sustained oxidative/nitrosative stress (increase in MDA and NO metabolites).

According to the results on ECNs toxicity, we found no differences in the survival of the two alveolar basal epithelial (A549) or brain microglial (BV-2) cell lines. This finding is of relevance since it has been shown that ECNs administered in vivo are capable to cross the blood brain barrier (BBB)^37^. Therefore, it is conceivable that repeated administration of ECNs, particularly under conditions of transitory or permanent BBB breakdown, such as in brain ischemia^38^, in traumatic brain injury^39^ or in various chronic neurodegenerative disorders^40^, may produce accumulation in the brain tissue at levels capable to cause cell death. In this context, we found that the surrogate of LS DPPC:POPG(7:3) was scarcely effective in increasing survival of alveolar basal epithelial and microglial cells treated with ECNs, unless challenge with nanoparticles was carried out at high ECN concentrations (Fig. 1).

On the basis of the change in cell survival to increasing ECN concentrations, we were interested in determining the effects of the maximal non-cyctotoxic dose of ECNs on various aspects of cell functions, including oxidative/nitrosative stress, energy metabolism, and mitochondrial phosphorylating capacity. Our results indicate that this ECN concentration (2 µg/mL) stimulates overproduction of NO (Fig. 2) and ROS (Fig. 3) in both cell lines. As expected, BV-2 cells (brain macrophages) showed higher NO production than A549 cells (alveolar basal epithelial cells). In both cell lines, results with nitric oxide synthase inhibitors (Supplementary Figure 4) clearly showed that NO production mostly occurred through iNOS activation. Both NO and ROS production were effectively counteracted by the treatment with the antioxidants *N*-acetyl-L-histidine and carnosine (Fig. 3). This finding is in accordance with previous data showing induction of ROS generation, increased cytoplasmic Ca^2+^ content, production of TNF-α, and enhanced caspase-3 activity, when using cationic nanoparticles (Si and Ge nanoparticles) for the treatment of rat alveolar macrophage (NR8383) and human colonic adenocarcinoma (Caco-2) cells^41^. Additionally, Pattani et al. reported the in vitro and in vivo effects of chitosan nanoparticles on NO production, IL-6 gene expression, and lymphocyte proliferation^42^. A dose-dependent increase in NO production was observed in peripheral blood mononuclear cells accompanied by lymphocyte proliferation. In the same study, the increase in NO production was also showed in vivo in a wound healing model. On the other hand, divergent results indicating that cerium oxide (CeO_2_) and yttrium oxide (Y_2_O_3_) nanoparticles decrease oxidative stress-induced apoptosis in isolated rat pancreatic islets exposed to hydrogen peroxide were proved by Hosseini et al.^43^. It is worthwhile recalling that our results, demonstrating sustained oxidative/nitrosative stress caused by challenge of alveolar basal epithelial and brain microglia cells with non-cytotoxic ECN levels, were obtained either by directly measuring NO and ROS formation using advanced microfluidic techniques, or by measuring stable end-products of NO metabolism (nitrite and nitrate) and of ROS-mediated lipid peroxidation (MDA) using well established HPLC methods. Furthermore, these data were strongly corroborated by the significant GSH depletion determined in same cell extracts (Table 4).

The experimental design we used in this study allowed to evidence that non-cytotoxic ECN dose was however capable to produce negative effects on fundamental metabolic functions connected to energy metabolism and mitochondrial functions. In particular, we observed an ATP decline by 23.5 and 29.1% in alveolar basal epithelial and brain microglial cells, respectively, that was accompanied by increase in ADP and AMP with consequent decrease in the value of the ECP. This indicates a state of energy penalty caused by imbalance in the main mitochondrial function that is to provide adequate ATP supply to ensure all the energy consuming reactions within cells. Malfunctioning of mitochondria was clearly evidenced by the decrease in the ATP/ADP ratio (Table 1), which is considered a good indicator of the mitochondrial phosphorylating capacity^32, 44^. The imbalance in energy metabolism caused by mitochondrial malfunctioning observed in our experiments was strongly reinforced by data referring to the decrease in triphophate nucleotides GTP, UTP, and CTP, accompanied by the increase in their corresponding di- and monophosphate forms (Table 2), indicating a generalized depletion of high energy compounds leading to significant cell energy crisis. Under these conditions of energy penalty and mitochondrial malfunctioning, it has been demonstrated that the complex mechanisms of the mitochondrial quality control, involving a complex system of genes and proteins that regulate the life of these organelles^45, 46^, are shifted towards fission and mitophagy^47, 48^, two processes that irreversibly compromise cell metabolism.

These findings are in line with results reported by Cui et al. who demonstrated in *E. coli* the ability of gold nanoparticles to collapse the mitochondrial membrane potential, to inhibit ATP synthase activities decreasing the ATP level, and to inhibit the binding of ribosome subunit to tRNA indicating a collapse of biological process^49^. Cell energy dysmetabolism along with the production of pro-inflammatory cytokines and cytotoxicity were also observed when using surface-functionalized silicon and germanium nanoparticles^41^. However, opposite results were reported by Suh et al. who showed that gold nanoparticles are able to prevent antimycin-A induced mitochondrial membrane potential dissipation, complex IV inactivation, ATP loss, Cyt c release, cardiolipin peroxidation, and oxidant generation in MC3T3-E1 osteoblastic cells^50^.

Treatment with non-cytotoxic dose of ECNs also produced depletion of the nicotinic coenzyme pool (NAD^+^, NADH, NADP^+^, and NADPH) (Table 3), therefore decreasing the correct supply of reducing equivalents for the mitochondrial electron transfer chain, as well as compromising the efficiency of all the oxido-reductive reactions in which these coenzymes are involved. This phenomenon has been observed in various experimental conditions characterized by energy penalty, such as myocardial ischemia and reperfusion^51^ and traumatic brain injury^52, 53^. Results related to nicotinic coenzymes also indicate that non-cytotoxic ECNs dose produced a significant decrease in the NAD^+^/NADH ratio. From a biochemical point of view, this is equivalent to a decrease in the lactate/pyruvate ratio and indicates an increased rate of glycolysis. Compensatory higher glycolytic rate occurs either because of decreased oxygen availability or, if oxygen is maintained constant as in our experiments, because of impaired mitochondrial functions with the aim of limiting decrease in ATP supply.

According to the results of the present study it is now possible to affirm that ECNs, even when used at non-cytotoxic dose, may produce relevant biochemical alterations involving not only sustained oxidative/nitrosative stress, but also profound imbalance in energy metabolism and mitochondrial functions. Since ECNs (and carbon-based nanomaterials in general) might find biomedical applicability either as drug carriers^54^ or as antineoplastic agents^55^, these new information are of significant utility for both possibilities. In the first possible application, it should be taken in great account the negative activities on different cell functions of ECNs as potential damaging side-effects that might limit their use even at low dosage. In the latter possible application, the imbalance of cell metabolism might be crucial if ECNs were selectively directed toward cancer cells and used as disruptors of energy homeostasis and mitochondrial functions of the target cells. In this context, Das et al. demonstrated that PLGA-loaded-nano-apigenin are very potent inhibitors of skin tumorigenesis through various mechanisms including mitochondrial dysfunction, ROS production, antioxidant depletion, and apoptosis^55^.

## Conclusion

Even though further studies are needed to evaluate the molecular mechanisms behind the biochemical alterations observed upon nanoparticles addition to the cell cultures, our findings highlight the importance of investigating the modulation of energy metabolism as well as NO and ROS production at cellular level even at very low-dose, apparently non-toxic, nanoparticle concentrations. The present study indicates that a much better comprehension of the molecular and biochemical changes caused by the interaction of ECNs (and nanoparticles in general) with living cells is mandatory before these highly promising tools might find the correct application in the biomedical field.

## Materials and methods

### Materials and reagents

Alveolar basal epithelial A549 cells (ATCC^®^ CCL-185™), RPMI-1640 medium, trypsin-EDTA solution, fetal bovine serum, and penicillin/streptomycin antibiotic solution were purchased from American Type Culture Collection (ATCC, Manassas, VA, USA). Microglial BV-2 cells (ICLC ATL03001) were purchased from Interlab Cell Line Collection (ICLC, Genova, Italy). Ultrapure standards for HPLC, tetrabutylammonium hydroxide, MTT [3-(4,5-dimethylthiazol-2-yl)-2,5-diphenyltetrazolium bromide] tetrazolium salt, In Vitro Toxicology Assay Kit, lactic dehydrogenase based, Cell Proliferation Kit II (XTT), L-N^G^-nitroarginine methyl ester (L-NAME), N^G^-monomethyl-L-arginine (L-MMA), L-histidine, β-alanine, L-carnosine, Griess reagent (modified), anhydrous dimethyl sulfoxide, potassium chloride, sodium dodecyl sulfate (SDS), phosphate-buffered saline (PBS), trypan blue solution, and sodium nitrite were all supplied by Sigma-Aldrich (St. Louis, MO, USA). HPLC-grade methanol, far-UV acetonitrile, and HPLC-grade chloroform were supplied by J. T. Baker Inc. (Phillipsburgh, NJ, USA). 2′,7′-dichlorodihydrofluorescein diacetate (H_2_DCFDA), phenol red-free RPMI-1640, *N*-acetyl-L-histidine, boric acid, sodium hydroxide, acetone, 2-propanol, and ethanol (95%) were obtained from Thermo Fisher Scientific Inc. (Pittsburgh, PA, USA). Polyethersulfone membrane (3 K) was purchased from VWR International (West Chester, PA, USA). C-Chip disposable hemocytometer was purchased from Bulldog Bio, Inc. (Portsmouth, NH, USA). Sylgard 184 polydimethylsiloxane (PDMS) prepolymer and curing agent were obtained from Ellsworth Adhesives (Germantown, WI, USA). Organic (chloroform) mixtures of the phospholipids, dipalmitoyl phosphatidylcholine (DPPC, 25 mg/ml) and 1-palmitoyl-2-oleoyl-sn-glyc-ero-3-phospho-(1′-rac-glycerol) (POPG, 10 mg/ml) used for this study were obtained from Avanti Polar Lipids Inc. (Alabaster, AL, USA). ECNs used in this research were obtained from Microdiamant (Lengwil, Switzerland). Nitrogen tanks for drying were supplied by Matheson Tri-Gas Inc. (Basking Ridge, NJ, USA). All water used was Ultrapure (18.3 MΩ cm) (Milli-Q Synthesis A10, Millipore, Burlington, MA, USA).

### Preparation of nanoparticle suspensions

Sample solutions of DPPC:POPG in 7:3 molar ratio were prepared in HPLC grade chloroform. This particular molar ratio was selected as a model mixture to emulate other synthetic LS compositions. The lipid mixtures were then dried with nitrogen and kept under vacuum overnight. The completely dried lipids were then suspended in PBS (pH 7.4) at a concentration of 10 mg/ml in a Precision water bath system (Thermo Fisher Scientific Inc., Pittsburgh, PA, USA) at 45 °C. This temperature was selected because the phase transition temperature of DPPC is 41 °C. ECN suspensions, prepared in PBS (pH 7.4), were sonicated for 2 h. Sonication is an essential step to prevent aggregation of the nanoparticle mixture. ECNs (1 wt. %) were then added to the phospholipid mixture for further studies.

### Nanoparticle characterization

Size distribution of ECNs, in water, was obtained using Dynamic Light Scattering (NanoBrook Omni, Brookhaven instruments Corporation, Holtsville, NY, USA) after 2 h of sonication. In water, the effective size was 219 nm with a polydispersity of 0.19. We monitored particle size for another half hour, which showed no change. A zeta potential of −28 mV was obtained in potassium chloride solution using the same instrument. TEM images of the ECNs originally suspended in water and organic solvent and AFM images of ECNs in the absence and presence of LS were previously reported by Chakraborty et al.^16^.

### Cell culture and treatment protocol

Alveolar basal epithelial A549 cells and microglial BV-2 cells were cultured in RPMI-1640 containing 10% (v/v) fetal bovine serum, streptomycin (0.3 mg mL^–1^), and penicillin (50 IU mL^–1^). The cells were cultured in 75 cm^2^ polystyrene culture flasks at a density of 5 × 10^6^ cells/flask, maintained in a humidified environment at 37 °C and 5% CO_2_, and passaged every 3–5 days, before they become confluent, to avoid overgrowth.

On the day of the experiment cells were harvested, counted, and plated at a density of 5 × 10^6^ cells/flask. Cells plated in 48-well plates (15 × 10^4^ cells/well) as well as the treatment with ECNs in presence of LS (DPPC:POPG(7:3)/ECNs) were used only for the determination of cell viability using the MTT assay. After 2 h cells were treated with nanoparticles (ECNs or DPPC:POPG(7:3)/ECNs) and incubated for 24 h in a humidified environment (37 °C and 5% CO_2_). For experiments carried out to investigate the antioxidants activity, cells were pre-treated with *N*-acetyl-L-histidine or carnosine (both of them at 10 mM final concentration) for 1 h prior to the treatment with nanoparticles. Resting cells were always used as control. At the end of 24 h incubation, to analyze intracellular ROS production, the cells were washed twice using 5 mL of cold 10 mM PBS at pH 7.4 and then incubated with phenol red-free RPMI-1640 containing H_2_DCFDA dye. Fresh stock dye solution of 10 mM H_2_DCFDA was prepared in 99% sterile dimethyl sulfoxide prior to each experiment. Next, 10 µL of this solution was added to each culture flask (10 μM H_2_DCFDA final concentration) and allowed to react for 60 min in a humidified environment (37 °C, 5% CO_2_). Cells were then washed using 5 mL of cold 10 mM PBS at pH 7.4 and harvested using 2.5 mL of trypsin-EDTA solution (0.25% Trypsin/0.53 mM EDTA in Hanks Balanced Salt Solution without calcium or magnesium). One hundred µL of the cell suspension was removed for cell counting before centrifuging the suspension at 125×*g* for 5 min at 4 °C. The supernatant was removed and the cell pellet was washed twice using cold 10 mM PBS at pH 7.4. Cells were lysed using 50 µL of pure ethanol and filtered by centrifugation at 18.690×*g* for 10 min at 4 °C in centrifuge tubes equipped with 3 kDa molecular weight cut-off filters. Ten microliter of this solution was added to 90 µL of running buffer consisting of 10 mM boric acid and 7.5 mM SDS at pH 9.2 (10% ethanol final concentration) and then analyzed with the microfluidic device^56^, while the stable nitric oxide end-product nitrite was measured using the Griess assay^57^.

The number of live cells was determined using the trypan blue exclusion assay. The cell suspension was diluted 1:1 to 1:3 (based on cell density) with 0.4% trypan blue solution. The cell density for each sample was determined using a C-Chip disposable hemocytometer.

### Total ROS detection using microchip electrophoresis with laser-induced fluorescence (ME-LIF)

The fabrication of hybrid polydimethylsiloxane-glass microfluidic devices to carry out the ME-LIF experiments has been described previously^56, 58^. Briefly, SU-8 10 negative photoresist was spin-coated onto a 4-inch diameter silicon wafer with a resulting thickness of 15 ± 1 mm employing a Cee 100 spin coater (Brewer Science Inc., Rolla, MO, USA). The obtained wafer was soft baked in two steps (pre-baked at 65 °C for 2 min and ramping to soft bake temperature of 95 °C for additional 5 min) using a hotplate (Thermo Scientific, Asheville, NC, USA). The selected software employed for microfluidic channel designs was AutoCad LT 2004 (Autodesk Inc., San Rafael, CA, USA). The designed geometry was printed onto a transparency film at a resolution of 50000 dpi (Infinite Graphics Inc., Minneapolis MN, USA). A transparency film mask was used to cover the coated wafer that was subsequently exposed (344 mJ cm^2^; 16 s) to UV light (i-line) (ABM Inc., San Jose, CA, USA). The wafer after UV exposure was post-baked in two steps (65 °C for 2 min and 95 °C for 10 min) and then developed in SU-8 developer, rinsed using 2-propanol (IPA), and dried with nitrogen. A “hard-bake” was performed at 180-200 °C for 2 h. A profilometer (Alpha Step-200, Tencor Instruments, Mountain View, CA, USA) was employed to confirm the thickness of the raised photoresist, which corresponds to the depth of the polydimethylsiloxane (PDMS) channels.

**Fig. 4:**
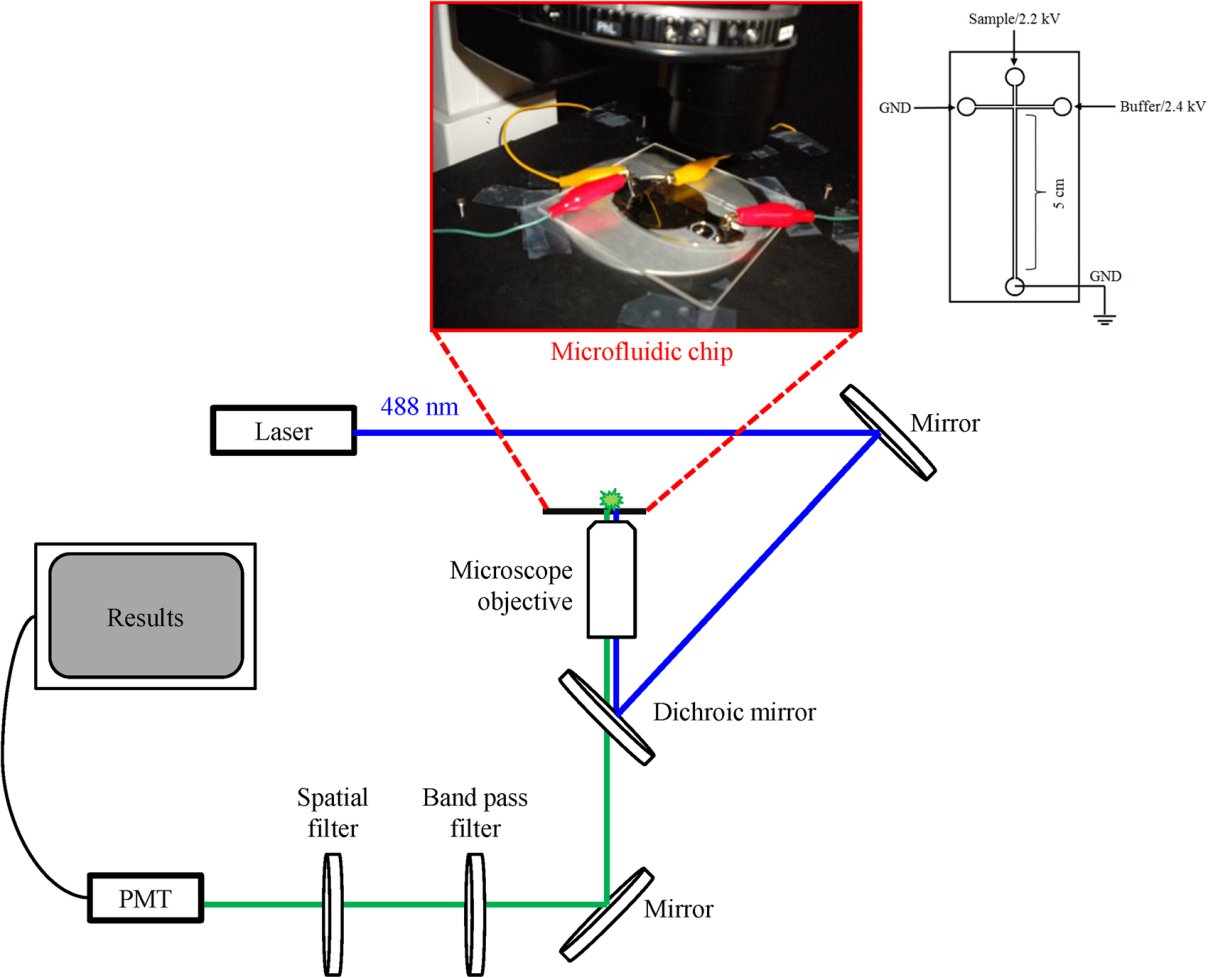
A schematic illustration of chip geometry (simple-T 5 cm microchip design) with applied voltages and ME-LIF setup. *GND* ground, *PMT* photomultiplier tubes

PDMS microchips were made by pouring PDMS prepolymer and curing agent (1:10 ratio) on the surface of the master that was subsequently placed in an oven at 70 °C overnight. We performed our experiments using a simple-T device with a 5 cm separation channel and 0.75 cm side arms (Fig. 4). A 4 mm biopsy punch (Harris Uni-core, Ted Pella Inc., Redding, CA, USA) was used to create the holes for all reservoirs (sample, buffer, ground). In order to complete the final hybrid PDMS-glass microchip device, the PDMS layer containing the embedded channels (presenting a width and a depth of 50 mm and 14 mm, respectively) was sealed to a borofloat glass plate.

Prior to analyzing each cell lysate, the PDMS-glass device was flushed with 0.1 M NaOH for 5 min, followed by a 5-min flush with running buffer (10 mM boric acid, 7.5 mM SDS at pH 9.2). Separations were performed in the normal polarity mode using a 30 kV high voltage power supply (Ultravolt, Ronkonkoma, NY, USA). For all separations, +2400 V was applied to the running buffer reservoir while +2200 V was applied to the sampling reservoir. The sample was introduced into the separation channel using a 1-s gated injection^59^. In order to remove any residual sample, the system was flushed for 60 s with running buffer after the analysis of each sample.

Excitation, detection, data acquisition, and data analysis were carried out using the same technologies and programs already described elsewhere^57^. A schematic illustration of chip geometry and ME-LIF setup is shown in Fig. 4.

### Indirect NO determination using the Griess assay

The stable NO end-product nitrite was measured using the Griess assay as previously described by Caruso et al^57^.

### MTT assay

The toxicity of the different preparation of nanoparticles (ECNs or DPPC:POPG(7:3)/ECNs) was measured through the determination of cell viability using the MTT [3-(4,5-dimethylthiazol-2-yl)-2,5-diphenyltetrazolium bromide] assay as previously reported^60^.

### HPLC analysis of metabolites

Both A549 and BV-2 cells were deproteinized after 24 h of incubation without and with non-toxic amount of nanoparticles. At the end of the incubation, cells were pelleted and washed twice with large volumes of cold 10 mM PBS at pH 7.4. After the second washing, cells were deproteinized according to the organic solvent deproteinization, suitable to measure acid labile and easily oxidizable compounds described in detail elsewhere^61^.

The simultaneous separation of high-energy phosphates (ATP, ADP, AMP, GTP, GDP, GMP, IMP, UTP, UDP, UMP, CTP, CDP, CMP), nicotinic coenzymes (NAD^+^, NADH, NADP^+^, NADPH), reduced glutathione (GSH), MDA, nitrite and nitrate in the protein-free cell extracts (200 μL) was carried out using previously established ion pairing HPLC methods^61, 62^, which utilize tetrabutylammonium hydroxide as the pairing reagent. Separation was obtained using a Hypersil C-18, 250 × 4.6 mm, 5 µm particle size column, provided with its own guard column (Thermo Fisher Scientific, Rodano, Milan, Italy). The HPLC apparatus consisted of a SpectraSYSTEM P4000 pump system (Thermo Fisher Scientific) and a highly-sensitive UV6000LP diode array detector (Thermo Fisher Scientific), equipped with 5 cm light path flow cell and set up between 200 and 300 nm wavelength. Assignment and calculations of the compounds of interest in chromatographic runs of cell extracts were performed by comparing retention times, absorption spectra, and area of the peaks (calculated at 260 nm wavelength in the case of high energy phosphates and nicotinic coenzymes, at 266 nm wavelength for MDA, or at 206 nm wavelength for GSH, nitrite and nitrate) of chromatographic runs of mixtures containing known concentrations of true ultrapure standard mixtures.

### Cell imaging

Images of A549 and BV-2 cells were obtained using an Accu-Scope microscope (Mel Sobel Microscopes Ltd, Hicksville, NY, USA) equipped with MicroPublisher 3.3 RTV camera (Qimaging, Surrey, BC, Canada). QCapture Pro 6 (Qimaging) was the selected software used for the image analysis.

### Statistical analysis

Normal data distribution in this work was determined using the Kolmogorov-Smirnov test. The within-group comparison was performed by one-way analysis of variance (ANOVA) while differences across groups were determined by two-way ANOVA. Fisher’s Protected Least Squares Differences was used as the post hoc test. Only two-tailed *p*-values less than 0.05 were considered statistically significant.

## Electronic supplementary material

Supplementary Figure and Table legends

Supplementary Figure 1

Supplementary Figure 2

Supplementary Figure 3

Supplementary Figure 4

Supplementary Table 1

Supplementary Table 2
