# Supplementary material for: Non-toxic engineered carbon nanodiamond concentrations induce oxidative/nitrosative stress, imbalance of energy metabolism, and mitochondrial dysfunction in microglial and alveolar basal epithelial cells
Source: Cell Death Dis. 2018 Feb 14;9(2):245. doi: 10.1038/s41419-018-0280-z (PMC5833425; doi:10.1038/s41419-018-0280-z)
Supplement: Supplementary file 1 — Supplementary Figure and Table legends [file 41419_2018_280_MOESM1_ESM.pdf]

Supplementary Figure and Table legends

Supplementary Figure 1. A representative picture at low (2 hours after seeding) (A-B) and high density (C-D) for both A549 and BV-2 cells employed in our study.

Supplementary Figure 2. LDH release by A549 and BV-2 cells treated with 2 µg/mL ECNs for 24 hours. Data are the mean of 3 independent experiments and are expressed as the percent variation with respect to the LDH release recorded in control cultures. Standard deviations are represented by vertical bars.

Supplementary Figure 3. Change in the cell viability caused by challenging for 24, 48, and 72 hours A549 and BV-2 cells with 2 µg/mL of ECNs. Data are the mean of 3 independent experiments and are expressed as the percent variation with respect to the viability recorded in control cultures.

Supplementary Figure 4. Intracellular and extracellular concentrations of NO (as determined by Griess assay) in resting and ECNs-stimulated (24 hours) A549 (Panel A) and BV-2 (Panel B) cells. Cells were pre-treated for 1 hour with L-NAME (500 µM) or L-MMA (1 mM) before starting treatment with 2 µg/mL of ECNs. Data are the mean of 3 independent experiments. Standard deviations are represented by vertical bars.

Supplementary Table 1. Effect of 24 hours of incubation of alveolar basal epithelial A549 and microglial BV-2 cells with 2 µg/mL of engineered carbon nanodiamonds (ECNs) on cell proliferation.

Supplementary Table 2. Effect of 24 hours of incubation of alveolar basal epithelial A549 and microglial BV-2 cells with 2 μg/mL of engineered carbon nanodiamonds (ECNs) on cell death.
