# Supplementary figures and images for: Non-toxic engineered carbon nanodiamond concentrations induce oxidative/nitrosative stress, imbalance of energy metabolism, and mitochondrial dysfunction in microglial and alveolar basal epithelial cells
Source: Cell Death Dis. 2018 Feb 14;9(2):245. doi: 10.1038/s41419-018-0280-z (PMC5833425; doi:10.1038/s41419-018-0280-z)

A

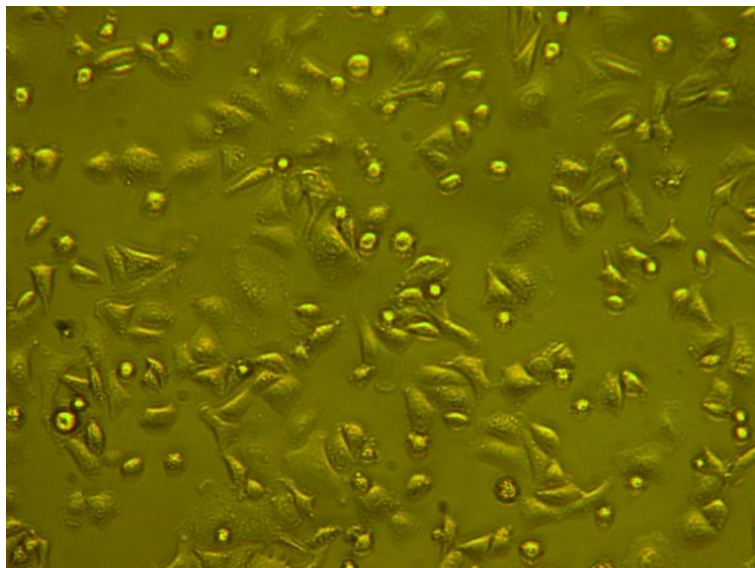

A549 cells – Low density

B

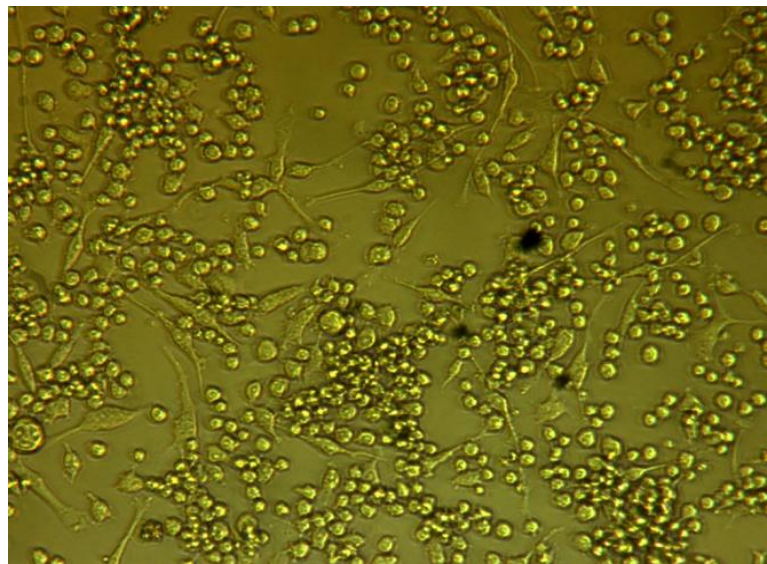

BV-2 cells – Low density

C

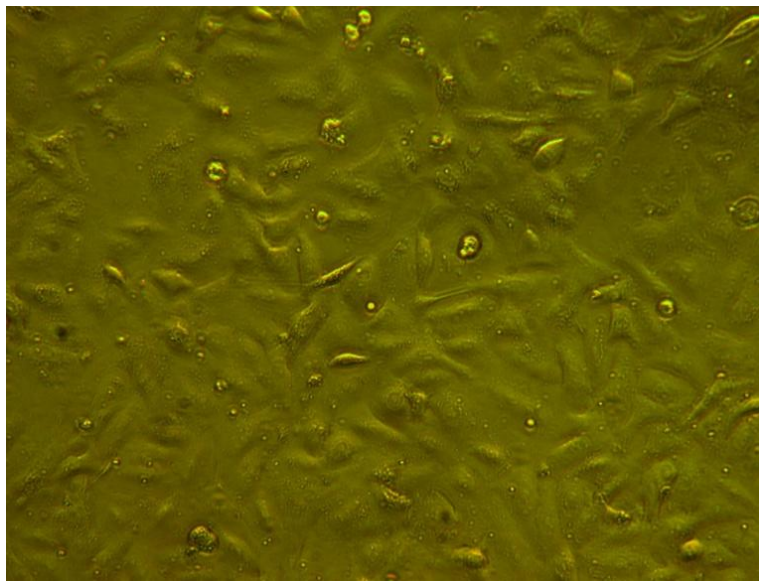

A549 cells – High density

D

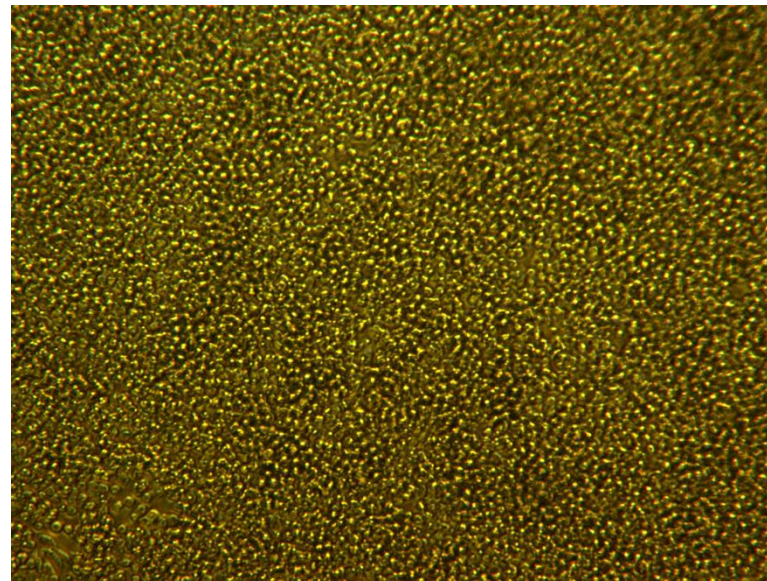

BV-2 cells – High density

Supplement: Supplementary file 2 — Supplementary Figure 1 [file 41419_2018_280_MOESM2_ESM.pdf]

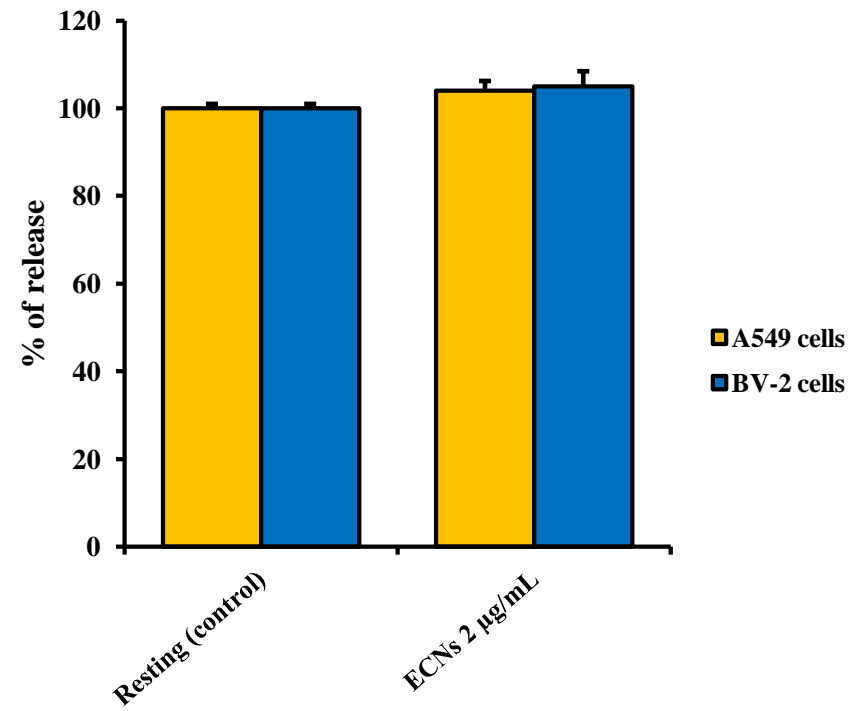

Supplement: Supplementary file 3 — Supplementary Figure 2 [file 41419_2018_280_MOESM3_ESM.pdf]

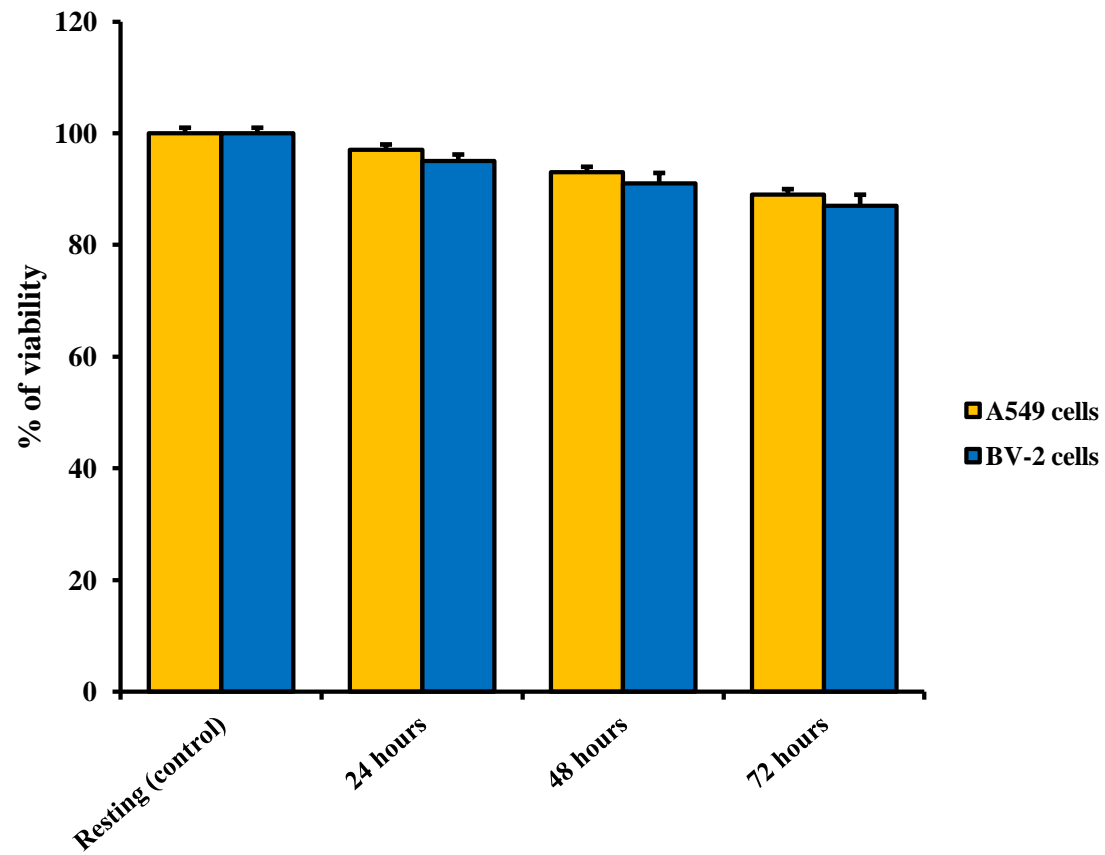

Supplement: Supplementary file 4 — Supplementary Figure 3 [file 41419_2018_280_MOESM4_ESM.pdf]

Panel A

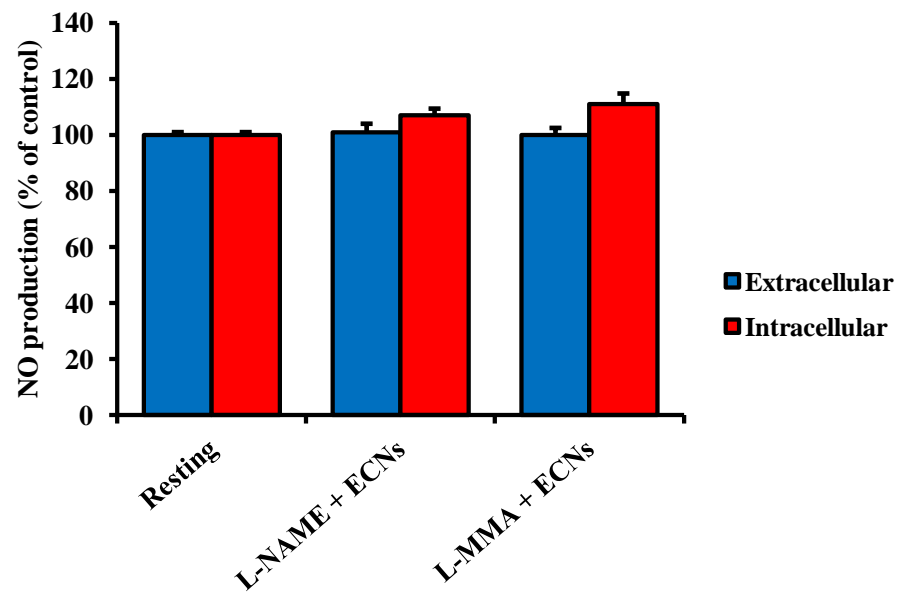

Panel B

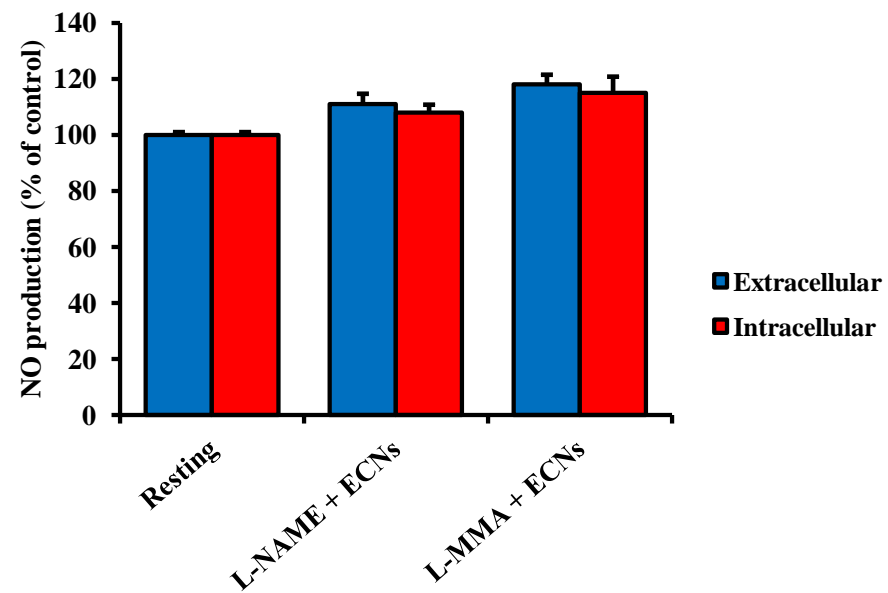

Supplement: Supplementary file 5 — Supplementary Figure 4 [file 41419_2018_280_MOESM5_ESM.pdf]
