# Supplementary material for: Non-toxic engineered carbon nanodiamond concentrations induce oxidative/nitrosative stress, imbalance of energy metabolism, and mitochondrial dysfunction in microglial and alveolar basal epithelial cells
Source: Cell Death Dis. 2018 Feb 14;9(2):245. doi: 10.1038/s41419-018-0280-z (PMC5833425; doi:10.1038/s41419-018-0280-z)
Supplement: Supplementary file 6 — Supplementary Table 1 [file 41419_2018_280_MOESM6_ESM.pdf]

Supplementary Table 1. Effect of 24 hours of incubation of alveolar basal epithelial A549 and microglial BV-2 cells with 2 μg/ml of engineered carbon nanodiamonds (ECNs) on cell proliferation.

**% of proliferation compared to resting cells**	
**A549 ECNs-treated**	**BV-2 ECNs-treated**
93.23<br>(5.95)	92.10<br>(5.12)

Values are the mean of four different experiments. Standard deviations are in parenthesis.
