# Supplementary material for: Non-toxic engineered carbon nanodiamond concentrations induce oxidative/nitrosative stress, imbalance of energy metabolism, and mitochondrial dysfunction in microglial and alveolar basal epithelial cells
Source: Cell Death Dis. 2018 Feb 14;9(2):245. doi: 10.1038/s41419-018-0280-z (PMC5833425; doi:10.1038/s41419-018-0280-z)
Supplement: Supplementary file 7 — Supplementary Table 2 [file 41419_2018_280_MOESM7_ESM.pdf]

Supplementary Table 2. Effect of 24 hours of incubation of alveolar basal epithelial A549 and microglial BV-2 cells with 2 μg/ml of engineered carbon nanodiamonds (ECNs) on cell death.

**% of cell death**			
**A549**		**BV-2**	
Resting	ECNs	Resting	ECNs
1.52<br>(0.39)	2.70<br>(0.45)	2.21<br>(1.82)	2.76<br>(1.20)

Values are the mean of four different experiments. Standard deviations are in parenthesis.
